# Circadian Clock Genes REV-ERBs Inhibits Granulosa Cells Apoptosis by Regulating Mitochondrial Biogenesis and Autophagy in Polycystic Ovary Syndrome

**DOI:** 10.3389/fcell.2021.658112

**Published:** 2021-08-05

**Authors:** Lihua Sun, Hui Tian, Songguo Xue, Hongjuan Ye, Xue Xue, Rongxiang Wang, Yu Liu, Caixia Zhang, Qiuju Chen, Shaorong Gao

**Affiliations:** ^1^Department of Reproductive Medicine Center, Shanghai East Hospital, School of Life Sciences and Technology, Tongji University, Shanghai, China; ^2^Department of Reproductive Medicine Center, Shanghai East Hospital, Tongji University School of Medicine, Shanghai, China; ^3^Department of Assisted Reproduction, Shanghai Ninth Peoples Hospital, Shanghai Jiaotong University School of Medicine, Shanghai, China; ^4^Institute for Regenerative Medicine, Shanghai East Hospital, Shanghai Key Laboratory of Signaling and Disease Research, Frontier Science Center for Stem Cell Research, School of Life Sciences and Technology, Tongji University, Shanghai, China

**Keywords:** polycystic ovary syndrome, REV-ERBs, mitochondrial biosynthesis, autophagy, follicular development

## Abstract

Polycystic ovary syndrome (PCOS) is an endocrinopathy with complex pathophysiology that is a common cause of anovulatory infertility in women. Although the disruption of circadian rhythms is indicated in PCOS, the role of the clock in the etiology of these pathologies has yet to be appreciated. The nuclear receptors REV-ERBα and REV-ERBβ are core modulators of the circadian clock and participate in the regulation of a diverse set of biological functions. However, in PCOS, the expression of REV-ERBs and their effects remain unclear. Here, we demonstrate that the levels of REV-ERBα and REV-ERBβ expression were lower in the granulosa cells of PCOS patients than in control subjects. *In vitro*, we found that the overexpression of REV-ERBα and REV-ERBβ, and their agonist SR9009, promoted the expression of mitochondrial biosynthesis genes PGC-1α, NRF1, and TFAM and inhibited autophagy in KGN cells. Our results also indicate that REV-ERBα and REV-ERBβ can inhibit apoptosis in granulosa cells and promote proliferation. Importantly, the REV-ERB agonist SR9009 ameliorates abnormal follicular development by promoting mitochondrial biosynthesis and inhibiting autophagy in a mouse PCOS model. This allows us to speculate that SR9009 has potential as a therapeutic agent for the treatment of PCOS.

## Introduction

Polycystic ovary syndrome (PCOS) is a common endocrinopathy with complex pathophysiology that can result in anovulatory infertility in women (Tanbo et al., 2018; Khan et al., 2019; Dehghani Firoozabadi et al., 2020). In addition to infertility, PCOS can give rise to hyperandrogenism, oligomenorrhea, hyperinsulinemia, chronic anovulation, and an increased risk of metabolic disorders such as type 2 diabetes and cardiovascular disease (Louwers and Laven, 2020; Osibogun et al., 2020). Critically, there has been a steady rise in the prevalence of PCOS worldwide among adult women (Manibalan et al., 2020). This has contributed to a need for further investigation of the mechanisms behind PCOS and for the development of efficient treatment strategies.

Granulosa cells have been identified to play a critical role in the follicle and oocyte development. These cells initially aid in the primary and secondary follicular growth and development by secreting nutrients and hormones that consequentially provide a comfortable microenvironment for the oocytes to undergo meiosis and maturation in the follicle (Ernst and Lykke-Hartmann, 2018; Komatsu and Masubuchi, 2018). Granulosa cell apoptosis results in reduced hormone biosynthesis in the ovary, which reduces growth and development of follicular oocytes and induces oocyte apoptosis (Prasad et al., 2016). In the ovary, energy required by these granulosa cells are supplied by subcellular mitochondrial organelles thus sustaining the production of vital hormones such as androgens and estrogens that are required for the follicle and oocyte development (Chowdhury et al., 2016; Liu et al., 2018). Interestingly, a defect in mitochondrial function and biogenesis could lead to consequences such as insulin resistance, hyperandrogenism, oxidative stress and glucose intolerance that are considered as key features in PCOS (Victor et al., 2009; Zhao et al., 2015). Additionally, low mitochondrial membrane potential (mtMP) is a critical characteristic that affects the quality of the oocyte thus acting as a key player affecting fertility outcomes (Safaei et al., 2020). Previously, a study has indicated that alterations in the mitochondrial biogenesis levels can give rise to hyperandrogenism and increase in the biogenesis of mitochondria can ameliorate PCOS by decreasing reactive oxygen species in granulosa cells, thus highlighting the potential role of mitochondria in PCOS (Safaei et al., 2020). Studies have shown that reduced mitochondrial biosynthesis promotes cell apoptosis (Diebold et al., 2015; Zhang et al., 2017; Wood Dos Santos et al., 2018).

Another key player that plays an important role in follicular development is the autophagy pathway. In a study by Duerrschmidt et al. (2006) a human cell model was used to study follicular atresia, wherein *in vitro* granulosa cells exposed to OxLDL showed enhanced autophagic cell death. Further, transmission electron microscopy (TEM) studies indicated an enhanced autophagy in the ovaries of women with PCOS along with elevated autophagic markers in granulosa cells in PCOS animal models (Li et al., 2018). Autophagy is induced mainly in granulosa cells during folliculogenesis and shows good correlation with apoptosis (Choi et al., 2010).

Recent studies have discovered that alterations in circadian regulation can trigger PCOS symptoms in animal models (Li et al., 2020). Biological clock genes have been identified to regulate and modulate the circadian rhythm (Zhou et al., 2019). A study indicated that one such biological clock genes, *BMAL1* (a cell-autonomous circadian clock oscillator) is highly downregulated in Granulosa cells (GCs) of PCOS patients, when compared to the non-PCOS group (Zhang et al., 2016). Another key circadian rhythm regulator is nuclear receptors REV-ERBα and REV-ERBβ (Bugge et al., 2012). A study by Woldt et al. (2013), indicated that REV-ERBα regulates the oxidative capacity of skeletal muscles by modulating the mitochondrial biogenesis and autophagy. Interestingly, other studies have indicated that REV-ERB agonists display anticancer properties by inhibiting lipogenesis and autophagy (Stujanna et al., 2017; Sulli et al., 2018). These studies imply that REV-ERBs may play a role in PCOS through the regulation of mitochondrial biogenesis and autophagy in granulocytes, however, the exact mechanism requires further study. In this study, we investigated the expression of REV-ERBs in follicular granulocytes from PCOS patients and elucidated the influence of the REV-ERBs and REV-ERB agonist, SR9009, on mitochondrial biosynthesis, and autophagy in KGN cells and in PCOS animal models.

## Materials and Methods

### Patients

Participants were recruited from women who were referred to Reproductive Medicine Center, Shanghai East Hospital. The diagnosis of PCOS was made according to the Rotterdam criteria. Patients who were included in the study presented two of the following: (i) oligomenorrhea and/or anovulation; (ii) hyperandrogenism; (iii) polycystic ovaries. Patients were excluded if they had any other condition involving the hypothalamus, pituitary, or ovary, such as hypothyroidism, or if they had received insulin or hormonal treatment in the preceding 3 months. The final study group included 30 PCOS patients and 30 control subjects. The control subjects had attended the center for *in vitro* fertilization because of male infertility. Each patient provided informed written consent and the study was approved by the review board of our institute.

### Isolation of Granulosa Cells

Follicular fluid and serum samples were collected from the patients and control subjects. Cells were collected by centrifugation (2,200 × *g* for 30 min at room temperature) and suspended in 1 ml phosphate-buffered saline (PBS). The suspension was added to Ficoll (Cytiva, Chicago, IL, United States) and centrifuged at 1,000 × *g* for 15 min at room temperature. Granulosa cells were recovered by aspiration and counted using a hemocytometer. Cells were stored at –80°.

### Cell Cultures and Treatment

KGN cells, a steroidogenic human granulosa cell-like tumor cell line (Nishi et al., 2001) were purchased from Procell Life Science and Technology Co., Ltd., and identified by short tandem repeat (STR) profiling. Cells were cultured in Dulbecco’s modified Eagle medium (DMEM)-F-12 with 10% fetal bovine serum and antibiotics (100 U/ml penicillin and 100 μg/ml streptomycin) in a 5% CO_2_ humidified atmosphere at 37°C. SR9009 (Xcess Biosciences, San Diego, CA, United States) was dissolved in DMSO and used at concentrations of 2.5 and 5 μM.

### Plasmids Constructs and Transductions

REV-ERBα and REV-ERBβ sequences were cloned into the p3XFlag-CMV-7.1 vector (Sigma-Aldrich, St. Louis, MO, United States). Interference of REV-ERBα and REV-ERBβ was conducted using the BLOCK-iT adenoviral RNAiexpression system (Invitrogen, Carlsbad, CA, United States) as described previously (Behrend et al., 2005). Vectors were transfected into cells using Lipofectamine (Invitrogen) following the manufacturer’s instructions.

### PCOS Rat Model

All the animal experiments were approved by Shanghai East Hospital and were carried out in accordance with the regulations set by Shanghai East Hospital. Ethical clearance was obtained from ethics committee of Shanghai East Hospital. Female SD rats (JRDUN Biotechnology, Shanghai, China) aged 3 weeks and weighing 50–55 g was maintained in a pathogen-free environment at 22°C with 33% humidity in 12 h light/12 h dark cycles. To establish the PCOS model, DHEA (Dehydroepiandrosterone, Sigma, Shanghai, China) (60 mg/kg BW s.c.) was injected daily for 21 days (Kim et al., 2018; Wen et al., 2020). PBS was substituted for DHEA in the control group.

### Experimental Design

Rats (*n* = 15) were randomly divided into control, PCOS, and PCOS + SR9009 groups (*n* = 5 per group). PCOS rats were treated with 100 mg/kg SR9009 by i.p. twice each day for 1 week and once a day for a further week. Blood samples and ovaries were collected for evaluation at the end of the experiment.

### Histopathological Examination of Ovary Tissues

Ovarian tissue was fixed in 4% paraformaldehyde for 48 h and dehydrated in a gradient of ethyl alcohol, soaked in xylene, and embedded in paraffin. The tissue samples were sliced into 5 mm sections and stained with H&E. Sections were then observed under a light microscope.

### TUNEL Assay

To assess apoptosis in granulosa and KGN cells a TUNEL assay kit (Roche Applied Science, Penzberg, Germany) was used following the manufacturer’s instructions. Fluorescence images were obtained using a laser-scanning confocal microscope (Leica, Wetzlar, Germany). The rate of apoptosis was calculated from 100 cells in six separate fields.

### Immunofluorescence Analysis

Granulosa and KGN cells were grown on coverslips and then fixed with 4% paraformaldehyde for 20 min at room temperature followed by 0.1% Triton X-100 for 15 min. Cells were then blocked-in goat serum and incubated with primary antibodies (Ki-67, Abcam, ab8191; PGC-1α, Proteintech, 66369-1-Ig; NRF1 Santa-Cruz, sc-28379; TFAM, Proteintech, 19998-1-AP; LC3 Abcam, ab48394) at 4°C overnight. Cells were then incubated with TRITC-conjugated goat anti-rabbit or anti-mouse IgG for 1 h and counterstained with DAPI. Immunofluorescence was detected and photographed using an inverted microscope.

### Transmission Electron Microscopy

TEM images of KGN cells were obtained by first fixing cells in 2.5% glutaraldehyde solution for 30 min at 4°C. Cells were then dehydrated with a gradient of ethyl alcohol, embedded in Araldite resin, and sliced into 60 nm sections. Sections were stained with uranyl acetate and lead citrate at room temperature for 15 min. The formation of autophagosomes and autolysosomes was observed in TEM images.

### qRT-PCR

Total RNA was extracted from samples using TRIzol Reagent (Invitrogen) following the manufacturer’s instructions. A First-Strand cDNA Synthesis Kit (Fermentas, Waltham, MA, United States) was used to synthesize cDNA. SYBR Green PCR Master Mix (ABI, Waltham, MA, United States) was used to conduct qPCR in a 7500 Real-Time PCR System (ABI) following the manufacturer’s recommendations. The primer sequences were as follows: REV-ERBα: F: CACATAC TTCCCACCATCACCT, R: ACAGTAGCACCATGCCGTTAAG; REV-ERBβ: F: TGTCTGTCCGTGGGAATGTC, R: ATGCGGC TCTGCTAAGGTGT; PGC-1α: F: GCCCCATGGATGAAG GGTACT, R: AGCGGCTGTTACTCTCTCTC; NRF1: F: CACAGAAAAGGTGCTCAAAGGA, R: TTTGGGTCACT CCGTGTTCC; TFAM: F: ACAGAGGTGGCTCAACAGCG, R: CGGAGATGAAGGGAAACCGC; GAPDH: F: CTGGAGAAAC CTGCCAAGTATG, R: GGTGGAAGAATGGGAGTTGCT. A mean cycle threshold was calculated for each sample and quantified in relation to GAPDH.

### mtDNA to nuDNA Ratio

Quantitative PCR of a mitochondrial gene mtNd1 (mitochondrially encoded NADH dehydrogenase (1) relative to a single copy nuclear gene, Cftr (Cystic fibrosis transmembrane conductance regulator) was used to measure mtDNA to nuDNA ratio. Total DNA was extracted from cells using a PureLink Genomic DNA Mini Kit (Invitrogen) and quantified using a NanoDrop 2000c Spectrophotometer (Thermo Scientific, Waltham, MA). Primer sequence targeting mtND1 and Cftr includes, mt-Nd1: F: TCCGAGCATCTTATCCACGC, R: GTATGGTGGTACTCCCGCTG, Cftr: F: ATGGTCCACAAT GAGCCCAG, R: GAACGAATGACTCTGCCCCT.

### Western Blot Analysis

Fresh ovarian tissues were washed in PBS and homogenized. The homogenate was centrifuged at 3,000 × *g* for 10 min and the supernatant was collected for analysis. Granulosa and KGN cells were lysed with cell lysis buffer (Nanjing KeyGen Biotech, Nanjing, China). The cell lysate was used for analysis. Protein content in samples was measured using a bicinchoninic acid assay kit. Equal amounts of proteins were separated with SDS-PAGE and transferred to polyvinylidene difluoride (PVDF) membranes (Millipore, Burlington, MA, United States). PVDF membranes were blocked with non-fat milk (5%) for 2 h and then incubated with primary antibodies (REV-ERBα Santa-Cruz, sc-393215, 1:100; REV-ERBβ Santa-Cruz, sc-398252,1:100; PGC-1α Proteintech, 66369-1-Ig, 1:1,000; NRF1 Santa-Cruz, sc-28379, 1:200; TFAM Proteintech, 19998-1-AP, 1:1,000; LC3 Abcam, ab51520, 1:3,000; p62 Abcam, ab83134, 1:500; GAPDH Abcam, ab8245, 1:500) overnight at 4°C. Finally, membranes were probed with horseradish peroxidase-conjugated goat anti-rabbit or anti-mouse secondary antibody and immunoreactivity was detected by enhanced chemiluminescence.

### Statistical Analysis

All data are expressed as mean ± SEM. Statistical analysis was performed using GraphPad Prism6 software (San Diego, CA, United States). For normally distributed data, the significance of differences was determined using a *t*-test or one-way factorial analysis of variance. Some data were also analyzed using SPSS Software version 16.0 (Chicago, IL, United States). *P* < 0.05 was considered to indicate a statistically significant difference.

## Results

### REV-ERBs Expression Is Downregulated in Granulocytes of PCOS Patients

**FIGURE 1:**
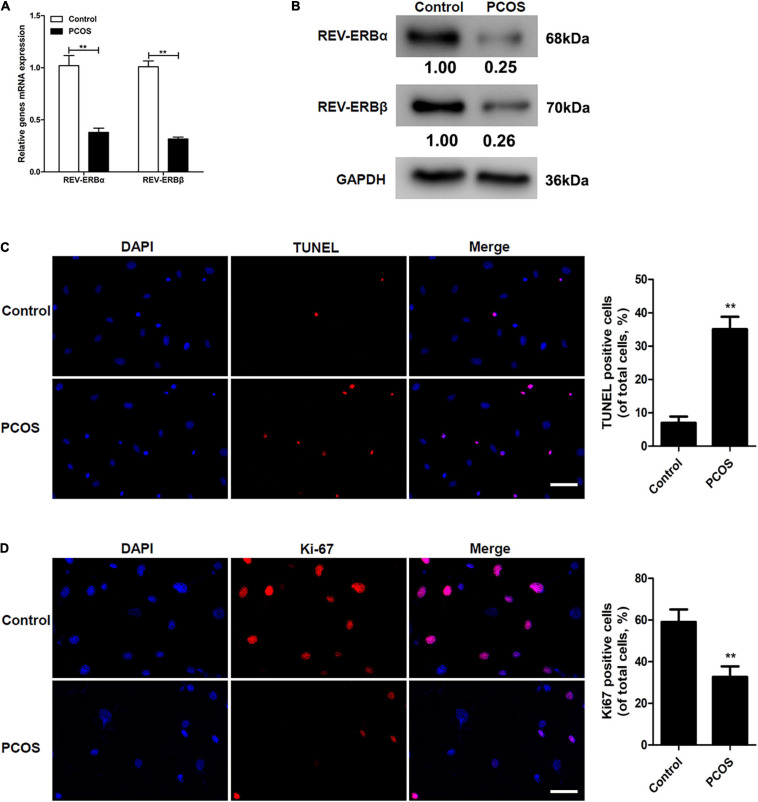
Comparison of REV-ERBs in the granulosa cells of PCOS patients. **(A,B)** REV-ERB levels in granulosa cells were detected by qRT-PCR and western blot analysis. **(C)** Granulosa cell apoptosis assayed by TUNEL, and the percentage of positive cells. **(D)** Granulosa cell proliferation (Ki67-positivity) was assessed by immunofluorescence, and the percentage of positive cells. Scale bars: 50 μm. Blue fluorescence represents DAPI staining; Red fluorescence represents Ki-67 expression. Data are presented as means ± SEM. **P < 0.01 vs. control.

To determine if the expression of REV-ERBs was altered in PCOS, we first determined the level of REV-ERBs expression in the granulosa cells of patients with PCOS compared to control subjects. The mRNA expression and protein levels of REV-ERBα and REV-ERBβ were significantly lower in the granulosa cells of patients with PCOS than in the cells of control subjects (Figures 1A,B). Simultaneously, we observed that there was an increase in TUNEL-positive cells in patients with PCOS compared to that of the control (Figure 1C). Contrastingly, levels of the cell proliferation marker Ki67 were lower in granulosa cells of patients with PCOS than in the control (Figure 1D). These results indicate that down-regulated REV-ERBs may be associated with apoptosis in the granulosa cells of patients with PCOS.

### Mitochondrial Biogenesis Is Driven by the Overexpression of REV-ERBs in KGN Cells

**FIGURE 2:**
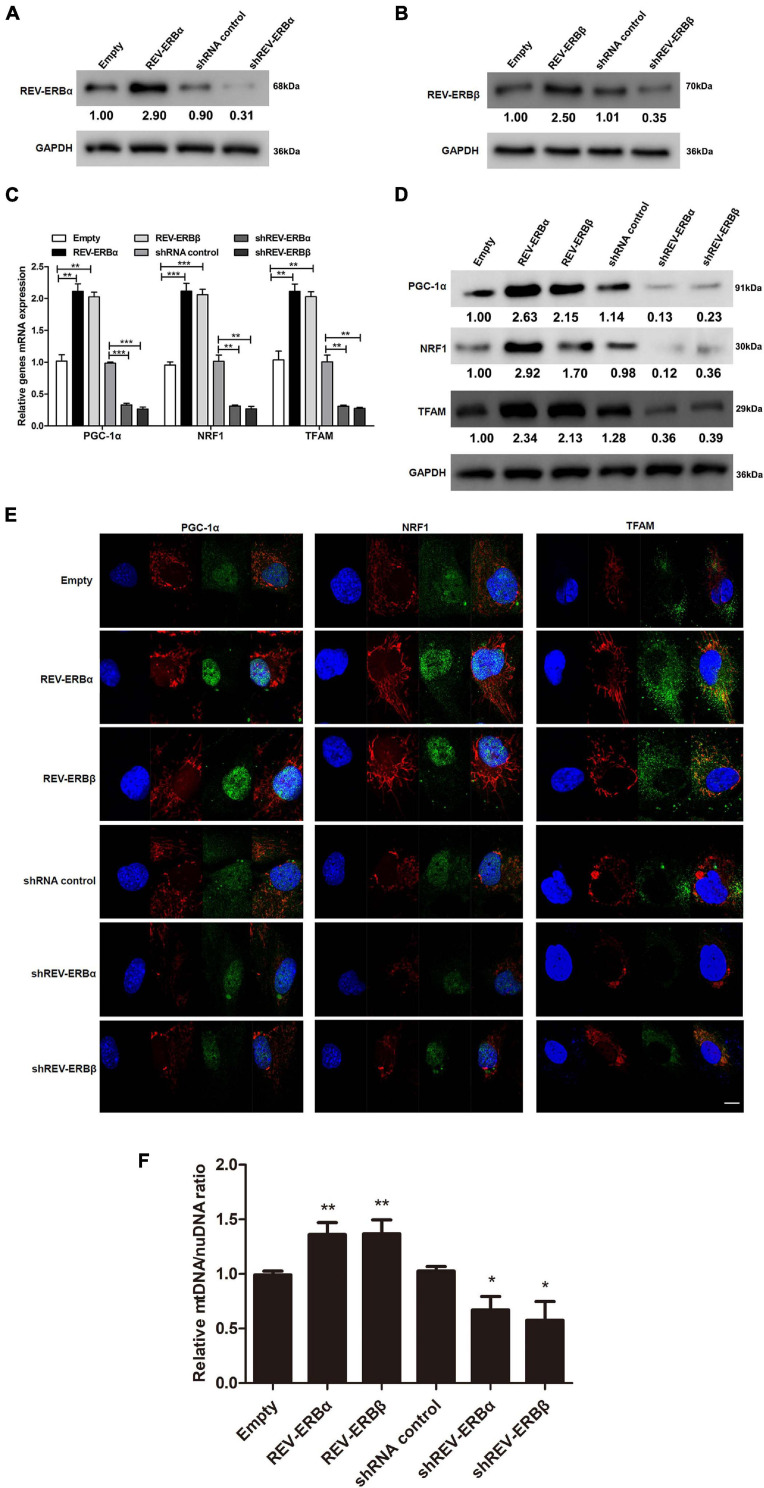
Overexpression of REV-ERBs drives mitochondrial biogenesis in KGN cells. KGN cells transfected with empty vector, REV-ERBα, REV-ERBβ, shRNA control, shREV-ERBα, or shREV-ERBβ plasmid for 48 h. **(A,B)** Overexpression or silencing efficiency was analyzed by measuring REV-ERBα and REV-ERBβ protein levels with western blotting. **(C,D)** Genes related to mitochondrial biogenesis levels in KGN cells were detected by qRT-PCR and western blotting. **(E)** Genes related to mitochondrial biogenesis in KGN cells were detected by immunofluorescence staining. Scale bars: 10 μm. Blue fluorescence represents DAPI staining; Red fluorescence represents the location of mitochondria, which is stained by Mitotracker; Green fluorescence represents the targeted protein expression; Merged images are yellow. **(F)** Mitochondrial to nuclear DNA ratio. Data are presented as means ± SEM. *P < 0.05, **P < 0.01, ***P < 0.001 vs. empty vector or shRNA control.

Previously, a study in C2C12 cell line had indicated that expression of REV-ERBs is essential for mitochondrial biogenesis (Amador et al., 2018) and mitochondrial biosynthesis is closely related to PCOS. Therefore, we assessed the influence of REV-ERBs on expression levels of genes associated with mitochondrial biogenesis in KGN cells (Nishi et al., 2001). Initially, REV-ERBα and REV-ERBβ either were overexpressed or silenced using overexpression or siRNA systems and their efficiency were confirmed using western blotting (Figures 2A,B). We next assessed the gene expression of PPAR gamma coactivator 1-alpha (PGC-1α), nuclear respiratory factor 1 (NRF1), and mitochondrial transcription factor A (TFAM) under conditions where REV-ERBs are overexpressed or silenced. PGC-1α is a transcriptional coactivator of genes that regulate mitochondrial biogenesis (Fernandez-Marcos and Auwerx, 2011), NRF1 encodes a transcription factor that activates genes involved in mitochondrial DNA transcription and replication (Wang et al., 2006), and TFAM binds mitochondrial promoter DNA to participate in mitochondrial genome replication (Campbell et al., 2012). The expression of all three mitochondrial biogenesis genes were elevated when REV-ERBα and REV-ERBβ were overexpressed, when compared to the empty vector control; However, when either REV-ERBα or REV-ERBβ was silenced the expression and protein levels of PGC-1α, NRF1, and TFAM were significantly lower than in the control (Figures 2C,D). To assess the changes in mitochondrial biogenesis, we used MitoTracker Red staining which revealed that indeed overexpression of REV-ERBs enhanced mitochondrial biogenesis as demonstrated by increased fluorescence compared to empty vector control (red fluorescence in Figure 2E). Meanwhile, we showed that overexpression of Rev-ERBS indeed promoted mitochondrial biogenesis by calculating the ratio of mtDNA to nuDNA (Figure 2F). Consequently, the expression of PGC-1α, NRF1, and TFAM (green fluorescence in Figure 2E) were also higher when REV-ERBα or REV-ERBβ were overexpressed. The suppression of REV-ERBα or REV-ERBβ led to a subsequent reduction in PGC-1α, NRF1, and TFAM expression and mitochondrial biogenesis. These data suggest that the REV-ERBs can drive mitochondrial biogenesis *in vitro*.

### REV-ERBs Inhibit Autophagy in KGN Cells

**FIGURE 3:**
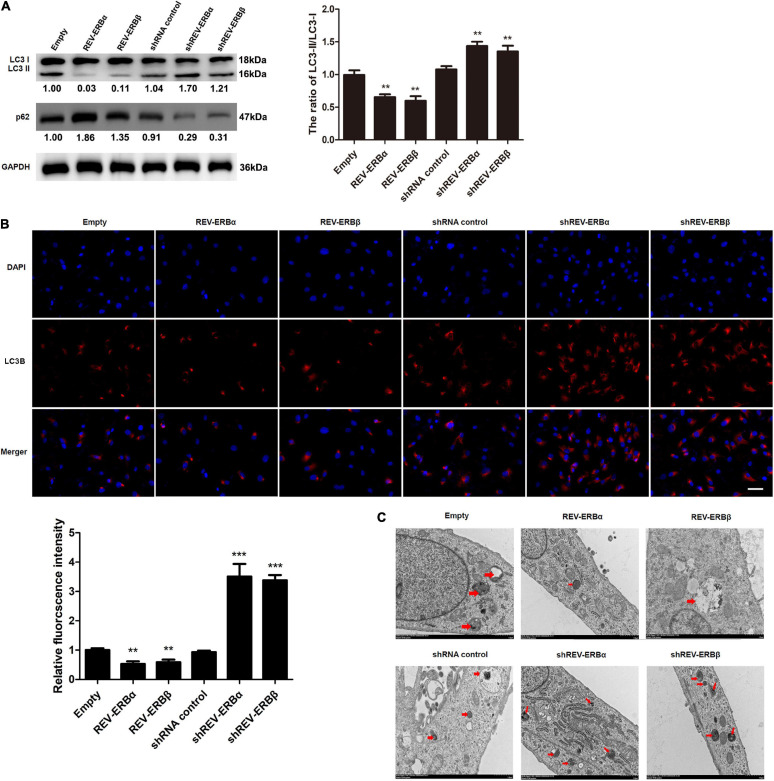
Overexpression of REV-ERBs inhibits autophagy in KGN cells. KGN cells transfected with empty vector, REV-ERBα, REV-ERBβ, shRNA control, shREV-ERBα, or shREV-ERBβ plasmids for 48 h. **(A)** The expression of LC3 and p62 in KGN cells was detected by western blotting. **(B)** The expression of LC3B in KGN cells was detected by immunofluorescence. Scale bars: 50 μm. Blue fluorescence represents DAPI staining; Red fluorescence represents LC3B expression. **(C)** Morphological observation of autophagy in KGN cells under transmission electron microscopy. Arrows indicate autophagosomes or autolysosomes. Scale bars: 1.0 μm. **P < 0.01, ***P < 0.001 vs. empty vector.

Having established an association between expression levels of REV-ERBs and mitochondrial biogenesis, we assessed the involvement of REV-ERBs in the autophagy of granulosa cells. To achieve this, we overexpressed or silenced REV-ERBs and we assessed the expression levels of microtubule-associated protein 1A/1B-light chain 3 (LC3) and p62 in KGN cells. During the initiation of autophagy a cytoplasmic form of LC3 (LC3-I) is conjugated to phosphatidylethanolamine (LC3-II) and is recruited to autophagosomal membranes (Tanida et al., 2008). The ubiquitin-associated protein p62 is a substrate of autophagy, linking autophagic molecules to the autophagic membrane by binding to LC3-II. Therefore, levels of p62 decrease during autophagy. We found that REV-ERBα and REV-ERBβ overexpression decreased the ratio of intracellular LC3-II/LC3-I, and increased p62 protein levels, indicating inhibition of autophagy; While REV-ERBα and REV-ERBβ silencing increased the ratio of LC3-II/LC3-I and decreased p62 protein levels, respectively, indicating a higher level of autophagy (Figure 3A). Immunofluorescence confirmed these results with the highest levels of LC3-II occurring when REV-ERBα and REV-ERBβ were downregulated and the lowest levels occurring when REV-ERBα and REV-ERBβ were upregulated (Figure 3B). TEM allowed the visualization of autophagosomes and autolysosomes in KGN cells (Figure 3C). The overexpression of REV-ERBα and REV-ERBβ resulted in fewer autophagosomes and autolysosomes in cells. These results demonstrate that REV-ERBs inhibits autophagy in granulosa cells.

### REV-ERBs Agonist SR9009 Drives Mitochondrial Biogenesis and Inhibits Autophagy in KGN Cells

**FIGURE 4:**
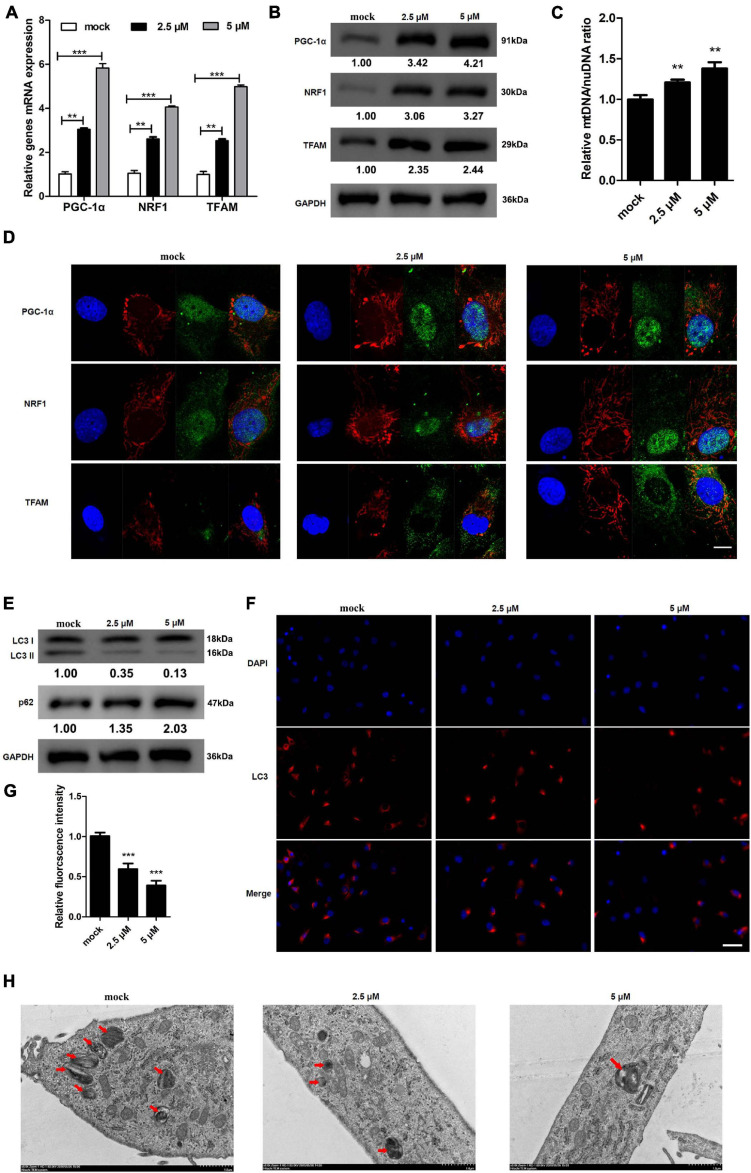
REV-ERB agonist SR9009 promotes mitochondrial biosynthesis and inhibits autophagy in KGN cells. KGN cells were treated with different concentrations of SR9009 (2.5, 5 μM) or mock-treated (mock) for 72 h. **(A,B)** Genes related to mitochondrial biogenesis levels in KGN cells treated with SR9009 were detected by qRT-PCR and western blotting. **(C)** Mitochondrial to nuclear DNA ratio. **(D)** Genes related to mitochondrial biogenesis protein levels in KGN cells treated with SR9009 were detected by immunofluorescence staining. Scale bars: 10 μm. Blue fluorescence represents DAPI staining; Red fluorescence represents the location of mitochondria, which is stained by Mitotracker; Green fluorescence represents targeted protein expression; Yellow indicates merged images. **(E)** The expression of LC3 and p62 in KGN cells treated with SR9009 were detected by western blotting. **(F)** The expression of LC3 in KGN cells were detected by immunofluorescence. Scale bars: 50 μm. Blue fluorescence represents DAPI staining; Red fluorescence represents LC3 expression. **(G)** Relative quantification of LC3 fluorescence intensity. **(H)** Morphological observation of autophagy in KGN cells treated with SR9009 under transmission electron microscopy. Scale bars: 1.0 μm. Data are presented as means ± SEM. **P < 0.01, ***P < 0.001 vs. mock.

SR9009 is a REV-ERB agonist that can modulate mitochondrial function (Stujanna et al., 2017). We investigated whether treating KGN cells with SR9009 could alter the expression of genes involved in mitochondrial biogenesis. Expression and protein levels of PGC-1α, NRF1, and TFAM increased dose-dependently in response to SR9009 (Figures 4A,B). These results were further confirmed using immunofluorescence staining and mtDNA/nuDNA ratio assay (Figures 4C,D). Mitochondrial biogenesis, the nuclear expression of PGC-1α and NRF1, and the cytoplasmic expression of TFAM increased with the increase in the dose of SR9009. We also explored the effects of different concentrations of SR9009 on autophagy in KGN cells. We found that as the concentration of SR9009 increased, autophagy in KGN cells decreased (Figures 4E–G). Further, TEM images indicated that as concentration of SR9009 increased, autophagy in KGN cells were clearly inhibited as indicated by decrease in autophagosomes and autolysosomes (Figure 4H). These results indicate that SR9009 promotes mitochondrial biosynthesis and inhibits autophagy in KGN cell.

### REV-ERBs and Its Agonist SR9009 Inhibit Apoptosis and Promote Proliferation of Granulosa Cells

**FIGURE 5:**
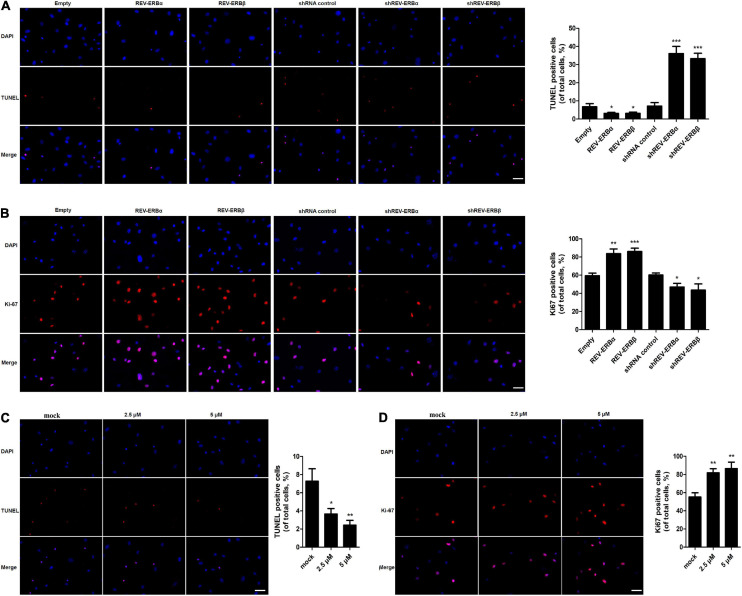
REV-ERBs and its agonist SR9009 inhibit apoptosis and promote the proliferation of KGN cells. KGN cells transfected with empty vector, REV-ERBα, REV-ERBβ, shRNA control, shREV-ERBα, or shREV-ERBβ plasmid for 48 h. **(A)** Granulosa cell apoptosis was assayed by TUNEL. Scale bars: 50 μm. Blue fluorescence represents DAPI staining; Red fluorescence represents TUNEL staining. **(B)** Granulosa cell proliferation (Ki67-positivity) was assessed by immunofluorescence. Scale bars: 50 μm. Blue fluorescence represents DAPI staining; Red fluorescence represents Ki-67 expression. KGN cells were treated with different concentrations of SR9009 (2.5, 5μM) or mock-treated (mock) for 72 h. **(C)** Granulosa cell apoptosis was assayed by TUNEL assay. Scale bars: 50 μm. Blue fluorescence represents DAPI staining; Red fluorescence represents TUNEL staining. **(D)** Granulosa cell proliferation (Ki67-positivity) was assessed by immunofluorescence. Scale bars: 50 μm. Blue fluorescence represents DAPI staining; Red fluorescence represents Ki-67 expression. *P < 0.05, **P < 0.01, ***P < 0.001 vs. empty vector or mock.

In addition, proliferation and apoptosis were assessed in KGN cells that were overexpressed or silenced for REV-ERBα and REV-ERBβ. Initially, we observed that cells overexpressing REV-ERBα and REV-ERBβ displayed fewer TUNEL-positive cells when compared to cells that were silenced for the REV-ERBs (Figure 5A). Consequently, we also observed an increase in proliferation in KGN cells overexpressing REV-ERBα and REV-ERBβ. Similar results were observed when cells were treated with SR9009. Increase in concentration of SR9009 led to a decrease in apoptosis along with an increase in proliferation in a dose-dependent manner (Figures 5C,D).

Overall, these results indicate that REV-ERBs and SR9009 lead to a decrease in apoptosis in KGN cells by promoting mitochondrial biogenesis and inhibition of autophagy.

### REV-ERB Agonist SR9009 Ameliorates Abnormal Follicular Development by Promoting Mitochondrial Biosynthesis and Inhibiting Autophagy *in vivo*

**FIGURE 6:**
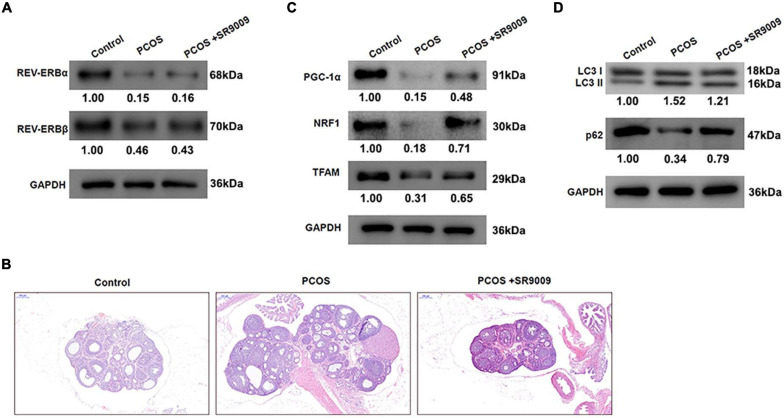
REV-ERBs agonist SR9009 prevents abnormal follicular development by regulating mitochondrial biosynthesis and autophagy in PCOS animal models. Female Sprague Dawley rats were injected with DHEA to construct the PCOS model, and treated with SR9009 or saline by i.p. twice per day for 1 week and then once a day for a further week. **(A)** The expression of REV-ERBs was detected by western blotting. **(B)** Representative images of H&E staining of ovarian tissues from rats in each group. Scale bars: 200 μm. **(C)** Genes related to mitochondrial biogenesis protein levels in ovarian tissues detected by western blotting. **(D)** The expression of LC3 and p62 was detected by western blotting.

To investigate whether similar observations could be made *in vivo*, female Sprague Dawley (SD) rats were injected daily with dehydroepiandrosterone (DHEA) to develop a PCOS model. Further, to assess the role of REV-ERBs on PCOS, rats were treated with SR9009 or saline twice each day for 1 week and then once a day for a further week. Initially, it was clear that the PCOS group rats expressed very less levels of REV-EBs, when compared to control groups. We also found that treatment with SR9009 had no effect on REV-ERBs levels, when compared to the PCOS groups (Figure 6A). Representative images of hematoxylin and eosin (H&E) stained ovarian tissues from rats indicate that PCOS rats have the typical large luminal space morphology; however, SR9009 improves the structure and morphology of ovarian tissue (Figure 6B). The protein levels of genes related to mitochondrial biogenesis were initially downregulated in the PCOS rats, however, SR9009 treatment upregulated these markers in the ovarian tissue (Figure 6C). Similarly, autophagic markers such as LC3 II was upregulated and p62 was downregulated in PCOS rats. However, SR9009 treatment reversed this effect indicating that indeed autophagy was reduced post SR9009 treatment (Figure 6D). These results demonstrate that SR9009 could inhibit autophagy, promote mitochondrial biogenesis and improve follicular development in a PCOS animal model.

## Discussion

PCOS is a disease with complex etiology affecting 6–7% of adult women (Franks, 2008). Recently, many studies have indicated that biological clock genes are associated with the development of PCOS (Li et al., 2011; Lim et al., 2019). A study by Li et al. (2011), identified that a polymorphism in Melatonin receptor 1A (*MTNR1A*) gene, a regulator of circadian rhythm, is associated with PCOS. Studies have identified a dysregulation in biological clock factors such cortisol (Lim et al., 2019), per2, and clock (Chen et al., 2016) to be highly correlated with an incidence of PCOS. However, till date the molecular mechanism associating biological genes to PCOS is still unclear. In this current study, we identified REV-ERBs to be highly downregulated in the granulocytes of PCOS patients (Figure 1). REV-ERBs are key circadian rhythm regulators that function as transcriptional repressors specifically in maintaining the transcriptional/translational feedback loops (Ikeda et al., 2019). REV-ERBs have functional roles in many metabolic, neuronal and inflammatory systems (Ikeda et al., 2019). However, its role and function in PCOS has not been well studied. Therefore, the current study aims at elucidating the role of REV-ERBs in PCOS. In PCOS, we observed that a decrease in REV-ERBs is positively associated with an increase in apoptosis and a decrease in proliferation in PCOS patient’s granulosa cells (Figure 1).

REV-ERBs have been identified to play critical roles in mitochondrial biogenesis (Amador et al., 2018). Studies observed that lack of REV-ERBα decreased mitochondrial biogenesis in skeletal muscle cells and isolated fibers (Woldt et al., 2013). Similarly, in our study, we observed that the lack of REV-ERBs significantly decreased the mitochondrial levels, as indicated by decreased Mitotracker staining and mtDNA/nuDNA ratio (Figure 2). Subsequently, we also observed a decrease in expression levels of mitochondrial biogenesis genes such as PGC-1α, NRF1, and TFAM in the absence of REV-ERBs. However, overexpression of REV-ERBs significantly increased mitochondrial biogenesis (Figure 2). This result was also observed in other studies where knockout of REV-ERBs in mice displayed decreased mitochondrial biogenesis as indicated by altered metabolic phenotype and nocturnal/sleep period (Amador et al., 2018). In skeletal muscles, this decrease in REV-ERBα have been associated with decreased AMPK activity and thereby decreased expression of PGC-1α, that contributes to decreased mitochondrial biogenesis and downregulated expression of other mitochondrial transcriptional factors (Woldt et al., 2013). In granulosa cells, abnormal mitochondrial function could lead to defective follicular development and maturation. Further, a defect in mitochondrial function and biogenesis could lead to other consequences that are considered as key features in PCOS (Victor et al., 2009; Zhao et al., 2015).

REV-ERBs have also been associated with autophagy. Autophagy is a key cellular process that maintains appropriate clearance of proteins and organelles under specific regulated stimulus (Mihaylova and Shaw, 2011). Regulation of autophagy is complex and any defect in such processes could lead to markedly grave consequences (Kroemer et al., 2010). In this study, we observed that lack of REV-ERBs is associated with an increase in autophagy, as indicated by increased levels of LC3-II and decreased levels of p62 (Figure 3). However, this defect in autophagy could be rescued by overexpression of REV-ERBs (Figure 3). Previous studies have indicated that REV-ERBα has the capacity to regulate autophagy by modulating genes involved in autophagosomal and lysosomal processes (Woldt et al., 2013). Similar to our results, absence of REV-ERBα seems to specifically increase the maturation of LC3-II from LC3-I in skeletal muscles (Woldt et al., 2013). Other studies have indicated that REV-ERBα, importantly also decreases mitophagy by repressing genes such as *Park2* which primarily functions by migrating to mitochondria and inducing mitophagy (Narendra et al., 2008). Additionally, REV-ERBα also seems to regulate *Ulk1* which is a known activator of mitophagy (Joo et al., 2011). This regulation of REV-ERBs on mitophagy further allows the maintenance of functional mitochondrial levels (Woldt et al., 2013). One potential mechanism by which REV-ERBs seem to achieve this inhibition/regulation of autophagy is by directly binding to these autophagy genes in their regulatory regions. This repression is achieved through decreased acetylation in the H3K27 and H3K9 sites (Woldt et al., 2013). Autophagy appears to affect granulosa cells in PCOS by two ways, primarily autophagy affects development of oocytes and hence the survival of granulosa cells. Secondarily, this causes inhibition of meiosis in oocytes and granulosa cells thereby decreasing maturation of the egg (Roche et al., 2016; Li et al., 2018). Hence, it is evident that regulation of mitochondrial and autophagy levels is essential for appropriate maturation and development of granulosa cells and a defect in any of these processes could lead to abnormal follicular development. We further used REV-ERB agonist SR9009, which confirmed that indeed upregulation of REV-ERB activity subsequently decreased autophagy and increased mitochondrial biogenesis in PCOS using both *in vitro* and *in vivo* animal model (Figures 4–6).

To our knowledge, this is the first study to elucidate the role of REV-ERBs in PCOS. This study also has allowed a deeper understanding of the mechanism behind PCOS, thus allowing identification of novel therapeutic targets for the treatment of PCOS. Additionally, SR9009’s role in rescuing/decreasing the deleterious effects of PCOS could allow development of potential treatment strategies against PCOS.

## Data Availability Statement

All datasets presented in this study are included in the article/Supplementary Material.

## Ethics Statement

The studies involving human participants were reviewed and approved by the Reproductive Medicine Center, Shanghai East Hospital. The patients/participants provided their written informed consent to participate in this study. The animal study was reviewed and approved by Shanghai East Hospital.

## Author Contributions

LS: conceptualization, data curation, analysis and interpretation, writing - review and editing, funding acquisition, and project administration. HT and SX: conceptualization, data curation, and roles or writing - original draft. HY: methodology, software, and validation. XX: methodology and validation. RW: investigation and methodology. YL and CZ: data curation and methodology. QC: conceptualization, writing - review and editing, funding acquisition, and project administration. SG: conceptualization, data analysis and interpretation, writing - review and editing, funding acquisition, and project administration. All authors contributed to the article and approved the submitted version.

## Conflict of Interest

The authors declare that the research was conducted in the absence of any commercial or financial relationships that could be construed as a potential conflict of interest.

## Publisher’s Note

All claims expressed in this article are solely those of the authors and do not necessarily represent those of their affiliated organizations, or those of the publisher, the editors and the reviewers. Any product that may be evaluated in this article, or claim that may be made by its manufacturer, is not guaranteed or endorsed by the publisher.

## Supplementary Material

Supplementary Figure 1 Comparison of Clock in the granulosa cells of PCOS patients. **(A,B)** REV-ERB levels in granulosa cells were detected by qRT-PCR and western blot analysis.

Supplementary Figure 2 qPCR detected the REV-ERBs gene and its downstream genes PGC-1α, TFAM, and NRF1 were expressed periodically in granulosa cells, and Clock gene was used as positive control.
