# Supplementary figures and images for: Circadian Clock Genes REV-ERBs Inhibits Granulosa Cells Apoptosis by Regulating Mitochondrial Biogenesis and Autophagy in Polycystic Ovary Syndrome
Source: Front Cell Dev Biol. 2021 Aug 5;9:658112. doi: 10.3389/fcell.2021.658112 (PMC8374745; doi:10.3389/fcell.2021.658112)

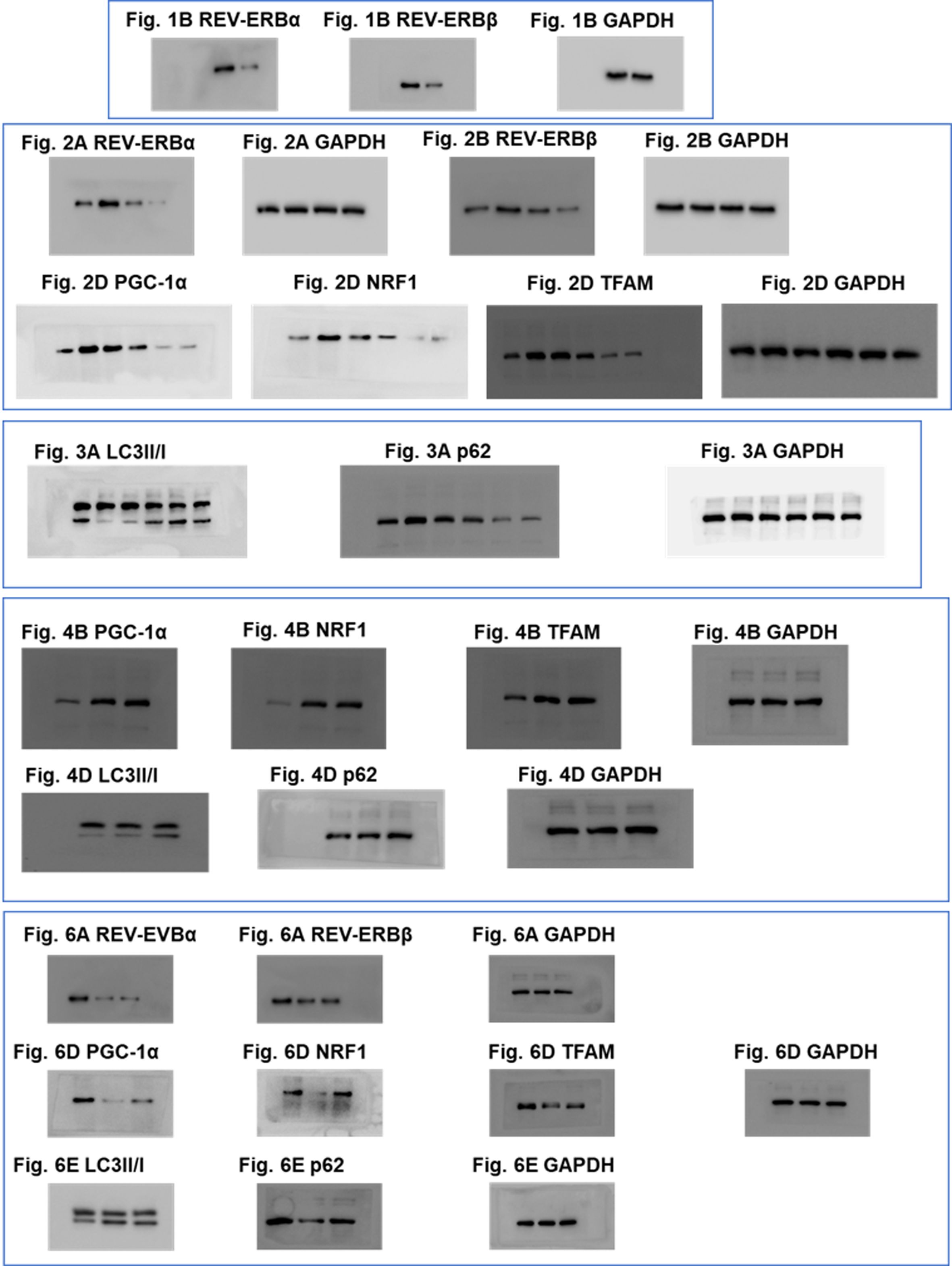

Supplement: Supplementary Figure 1 — Comparison of Clock in the granulosa cells of PCOS patients. (A,B) REV-ERB levels in granulosa cells were detected by qRT-PCR and western blot analysis. [file Image_1.JPEG]

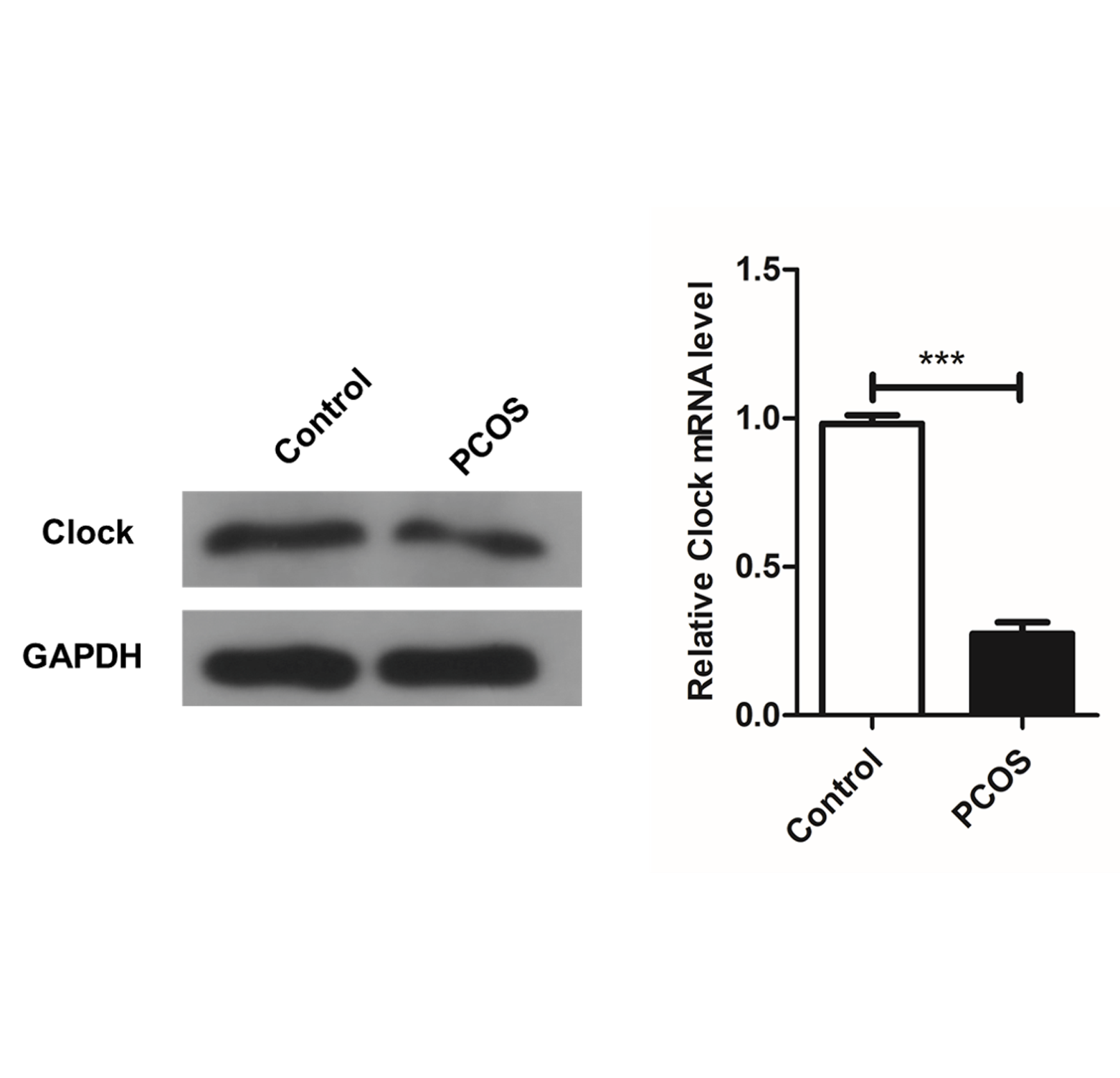

Supplement: Supplementary Figure 2 — qPCR detected the REV-ERBs gene and its downstream genes PGC-1α, TFAM, and NRF1 were expressed periodically in granulosa cells, and Clock gene was used as positive control. [file Image_2.TIF]

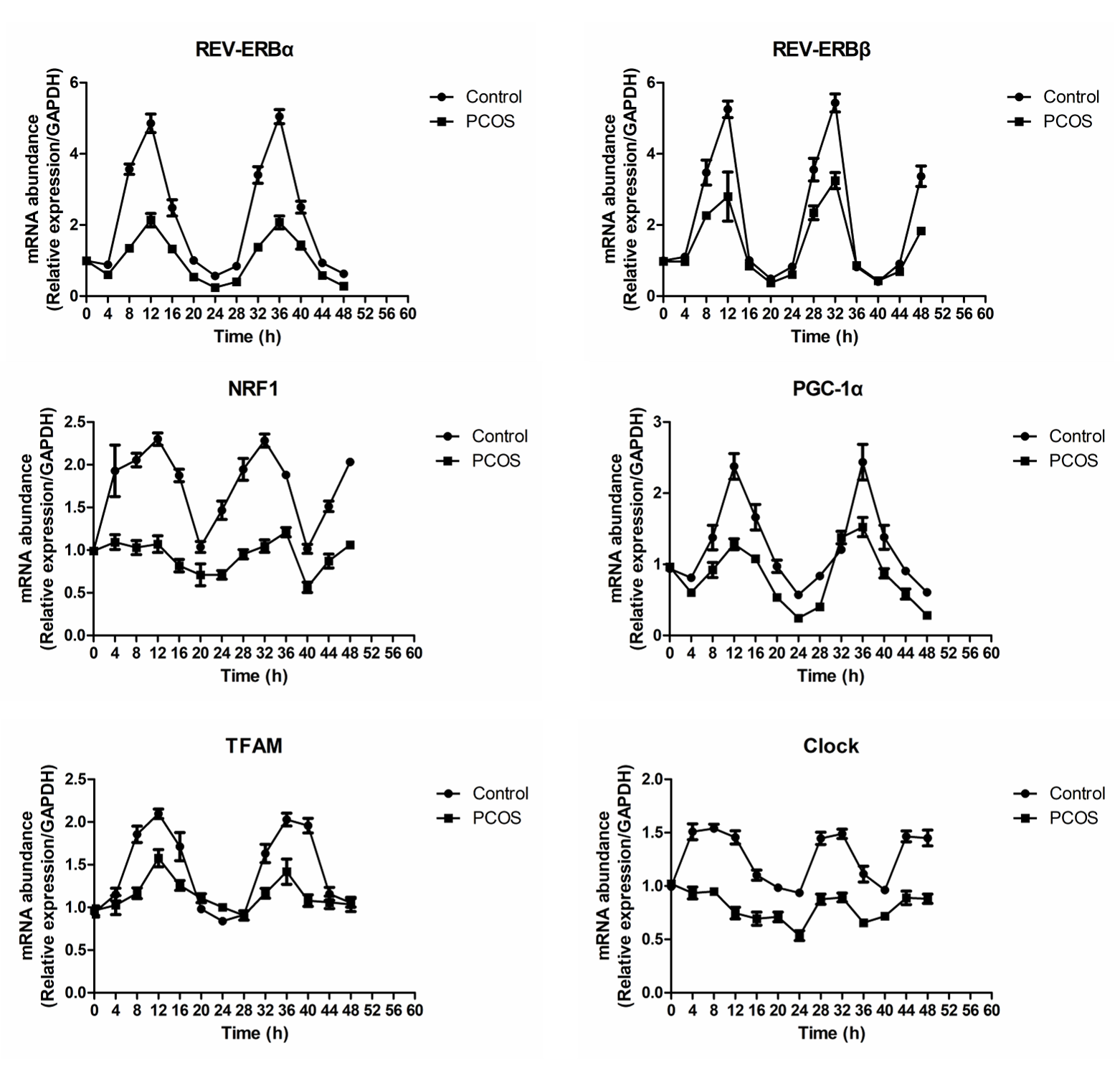

Supplement: Supplementary file 3 [file Image_3.TIF]
